# Depression, perceived stress, social support, substance use and related sociodemographic risk factors in medical school residents in Nairobi, Kenya

**DOI:** 10.1192/bjo.2021.181

**Published:** 2021-06-18

**Authors:** Sayed Shah Nur Hussein Shah, Ahmed Laving, Violet Okech-Helu, Manasi Kumar

**Affiliations:** 1 Kiambu Referral Hospital; 2 Senior lecturer, University of Nairobi; 3 Consultant Psychiatrist, Kenyatta National Hospital

## Abstract

**Aims:**

Little is known about mental health risk factors in medical residents and doctors in Sub-Saharan Africa. Residents are at a greater risk of developing depression, stress and substance abuse than the general public owing to the stressful nature of their medical training. Poor mental health in residents leads to decreased clinical efficiency and training satisfaction; it can also lead to substance dependence, self-harm and suicide.Our primary aim was to ascertain depression prevalence among medical residents in Kenya's largest national teaching and referral hospital. Secondary aims were to analyze how depression was associated with perceived stress, perceived social support, substance use, and educational environment.

**Method:**

Self report questionnaires were administered in this cross-sectional survey to 338 residents covering eight specialties (Medicine, Psychiatry, Paediatrics, Obstetrics/Gynaecology, Anaesthesia, General Surgery, Cardiothoracic Surgery, and Ear Nose Throat Surgery). In addition to key sociodemographics, the Centres for Epidemiology Depression Scale - Revised, Perceived Stress Scale, Multidimensional Scale of Perceived Social Support, Alcohol, Smoking and Substance Involvement Screening Test, and Postgraduate Hospital Educational Environment Measure were used.

**Result:**

The mean participant age was 31.8 years and 53.4% were males. Most residents (70.4%) reported mild/no depressive symptoms 12.7% had moderate, and 16.9% had severe symptoms.High social support (71.8%) and moderate stress (61.6%) were reported by most residents. Almost half (46.3%) rated their educational environment as being more positive than negative. Out of 238 respondents 11.3% were at moderate risk of health and other problems due to cocaine use, while 13.3% (out of 240 respondents) were at risk due to alcohol. On bivariate analyses, we found significant correlations between depression, perceived stress, substance use, perceived social support, and educational environment. Multivariate analysis revealed that depression was strongly associated with: fewer hours of sleep (β = -0.683, p = 0.002), high perceived stress (β = 0.709, p < 0.001) and low perceived social support (β = -2.19, p < 0.001).

**Conclusion:**

High perceived social support, low perceived stress, and less sleep were significantly associated with lower depression scores. A large proportion of residents were at risk of developing depression (29.6%). There were high levels of perceived social support (71.8%). A concerning proportion of residents used substances like alcohol and cocaine. This work is one of few that describe the mental health of an important and understudied population group in an LMIC. Priority must be given to protect and promote the mental health of such a vulnerable group.

Data collection and analysis were funded by Kenyatta National Hospital.

